# The kidney of late preterm infants

**DOI:** 10.1186/1824-7288-40-S2-A14

**Published:** 2014-10-09

**Authors:** Vassilios Fanos, Clara Gerosa, Claudia Fanni, Cristina Loddo, Melania Puddu, Giovanni Ottonello, Gavino Faa

**Affiliations:** 1 NICU, Neonatal Pathology, Puericulture Institute and Neonatal Section, AOU and University of Cagliari, Italy; 2 Institute of Pathology, AOU and University of Cagliari, Italy

## Introduction

The risk of morbidity in late preterm neonates varies greatly depending on gestational age: it is 1 out of 2 at 34 weeks, 1 out of 4 at 35 weeks and 1 out of 10 at 36 weeks. Very little is known about the renal pathology of these infants.

## Nephrogenesis in the late preterm

The process of nephron formation ceases between 34 and 36 weeks of gestation [1], the limit within which the term late preterm is applied.

In 1943, Potter and Thierstein examined the autopsies of 1000 fetuses and neonates and found the presence of the nephrogenic zone in 100% of 30-week fetuses, in about 80% of 34-week cases and in 30% of 36-week cases. They stated that in most of these neonates nephrogenesis had ceased at 35 weeks [2].

In 2008, Ferraz et al., on applying immunohistochemistry to the kidneys of 86 fetuses of different gestational ages, observed the disappearance of the nephrogenic zone in all fetuses above 35 weeks of gestational age [3].

**Figure 1:**
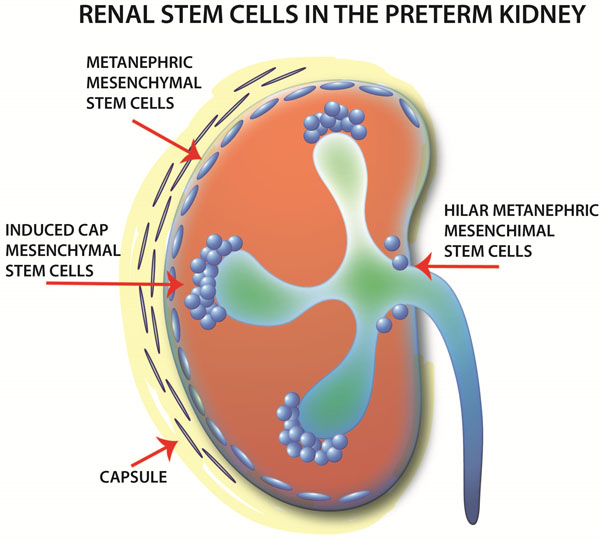
Zones where stem cells were found in the kidney of the late preterm infant (from Faa G et al. JPNIM 2014 in press, with permission)

On the contrary, Faa et al. found the presence of active nephrogenesis up to the 38th week [4]. It appears that in agreement with the data of Rodriguez et al. [5], the nephrogenetic process continues after preterm birth for a period of about 6 weeks; this window decreases further if the neonate develops acute renal injury or if he/she presents a intrauterine growth retardation. Stem cells are present in different parts of the neonatal kidney (Figure 1) [6]. A marked interindividual variability in the number of nephrons has been observed: 6 to 8 glomerular columns were present in late preterm infants (8 columns in Rodriguez' cases), but also in a large number of those up to 23 weeks.

## Renal function and pathology in the late preterm

Cuzzolin et al. studied 246 preterms divided into 4 groups based on gestational age (one of late preterms): the creatinemia values at birth were similar in the groups, with differences appearing from the 3rd and up to the 21st day of postnatal life [7,8].

No correlation between late preterm birth and the onset of renal pathologies was found shortly after or some time after delivery. This was confirmed by Picone's wide ranging study on 417 late-preterm infants [9].
